# What matters to me – a web-based preference elicitation tool for clients in long-term care: a user-centred design

**DOI:** 10.1186/s12911-020-1067-6

**Published:** 2020-03-17

**Authors:** Catharina M. van Leersum, Albine Moser, Ben van Steenkiste, Marion Reinartz, Esther Stoffers, Judith R. L. M. Wolf, Trudy van der Weijden

**Affiliations:** 1 Department of Family Medicine, CAPHRI School for Public Health and Primary Care, Maastricht University Medical Centre, P.O. Box 616, 6200 MD Maastricht, The Netherlands; 2 Research Centre for Autonomy and Participation of Persons with a Chronic Illness, Zuyd University of Applied Sciences, P.O. Box 550, 6400 AN Heerlen, The Netherlands; 3 Zorgbelang inclusief, P.O. Box 5310, 6802 EH Arnhem, The Netherlands; 4 Burgerkracht Limburg, P.O. Box 5185, 6130 PD Sittard, The Netherlands; 5 Impuls – Netherlands Center for Social Care Research, Radboud Institute for Health Sciences, Radboud University Medical Center, P.O. Box 9101, 6500 HB 117, Nijmegen, The Netherlands

**Keywords:** Preference elicitation, Long-term care, Patient preferences, User-Centred design, User requirements, Decision support techniques

## Abstract

**Background:**

During the process of decision-making for long-term care, clients are often dependent on informal support and available information about quality ratings of care services. However, clients do not take ratings into account when considering preferred care, and need assistance to understand their preferences. A tool to elicit preferences for long-term care could be beneficial. Therefore, the aim of this qualitative descriptive study is to understand the user requirements and develop a web-based preference elicitation tool for clients in need of long-term care.

**Methods:**

We applied a user-centred design in which end-users influence the development of the tool. The included end-users were clients, relatives, and healthcare professionals. Data collection took place between November 2017 and March 2018 by means of meetings with the development team consisting of four users, walkthrough interviews with 21 individual users, video-audio recordings, field notes, and observations during the use of the tool. Data were collected during three phases of iteration: Look and feel, Navigation, and Content. A deductive and inductive content analysis approach was used for data analysis.

**Results:**

The layout was considered accessible and easy during the Look and feel phase, and users asked for neutral images. Users found navigation easy, and expressed the need for concise and shorter text blocks. Users reached consensus about the categories of preferences, wished to adjust the content with propositions about well-being, and discussed linguistic difficulties.

**Conclusion:**

By incorporating the requirements of end-users, the user-centred design proved to be useful in progressing from the prototype to the finalized tool ‘What matters to me’. This tool may assist the elicitation of client’s preferences in their search for long-term care.

## Introduction

Preference elicitation is a requirement of decision support tools that assist clients faced with difficult decisions when in need of long-term care [1, 2]. Preference elicitation is difficult due to unknown outcomes of long-term care and non-quantifiable preferences, however, it could provide information on how someone rates and trades-off preferences [1, 3]. A preference elicitation tool could be beneficial during the process of consideration, because decisions on long-term care are made in an environment where the constraints and consequences of the decision are not precisely known [4]. To assist clients with their decision, act collaboratively, respect autonomy, and have an empathic approach, the collaborative deliberation model is developed [5]. In this model, five communicative steps are necessary: constructive interpersonal engagement, recognition of alternative actions, comparative learning, preference construction and elicitation, and preference integration [5]. The benefit of this model is the incorporation of preference elicitation in the communication. Currently, there are no tools available to assist clients during decision-making or preference elicitation for long-term care.

The aim of this qualitative descriptive study is to understand the user requirements and develop a web-based preference elicitation tool for clients in need of long-term care. Long-term care is regarded as care provided for at least six months for reasons of ageing, disability, chronic illness, or any situation that limits the ability to self-care and manage activities, e.g. washing, grocery shopping, or work [6]. Care can be provided in any setting, including home care, care facilities, or nursing homes [7]. In the Dutch context, long-term care is provided in four sectors: the nursing and care of elderly, mental healthcare, care of people with disabilities, and social care.

In the Netherlands, the healthcare policy aims to incorporate personal preferences with a freedom of choice into the decision-making process of clients in long-term care. The tool could become a means to help clients make a careful decision about services, caregivers, and care organizations by selecting preferences [8]. This might create a client-centred approach to decision-making [9]. The tool contains propositions with possible preferences that clients can rate on a 7-point Likert scale (see Additional file 1). Responses are visualized in the overview page of the tool. The overview page with client preferences is intended to be used as a preparation for discussion with long-term care professionals (such as independent care coordinators) about the client’s life and care. Independent care coordinators have the role of a decision counsellor and can support and facilitate the decision-making process with clients. Independent care coordinators share knowledge about the route a client could follow during decision-making and the options a client might have, and in some cases they support during consultations with care organizations.

Challenges in preference elicitation are volatility of preferences in time, dependence on the context, previous experiences, urgency of the decision, and personal goals [10, 11]. These aspects continuously influence personal preferences both consciously and unconsciously [12]. Without support to create an overview of preferences [13], the deliberation regarding long-term care is difficult, not always in line with personal expectations and values, and often leads to unfavourable choices that reduce quality of life [14]. Support is helpful, because clients are often not aware of all their preferences until they feel one of them has been infringed [13].

Preference elicitation mostly takes place in conversations within a client’s social network, using informal sources and experiences of others, but this is not enough and clients ask for assistance with decision-making [15]. Current assistance consists of information on standardized quality indicators of care and/or care organizations, such as team performance of professionals or number of reported incidents. However, it is known that these indicators do not influence decision-making [16]. When searching for long-term care, clients value issues such as travelling distance to relatives, the possibility of having a pet, a ‘click’ with caregivers, or eating in the company of others [14].

Clients experience difficulties in articulating their preferences and healthcare professionals often find it difficult to interpret these preferences [16]. There is an ongoing discussion among professionals regarding the need for tools that could support building conversations about preferences [17]. An example is the outcome prioritisation tool, which shows that professionals have a better understanding of the values of a client [18]. Other research shows that the use of a questionnaire in advance of consultations enhances communication and engagement [19]. Similar to decision-aid guidelines, these tools are meant to assist and not to replace consultations [20]. However, little is known about the content that would be relevant to such questionnaires. This study uses knowledge from qualitative research [14], and a user-centred design to develop a tool that is tailor-made together with end-users. The study is part of a larger project on assistance with the decision-making process of clients in four long-term care sectors in the Netherlands.

## Method

### Design

**Fig. 1:**
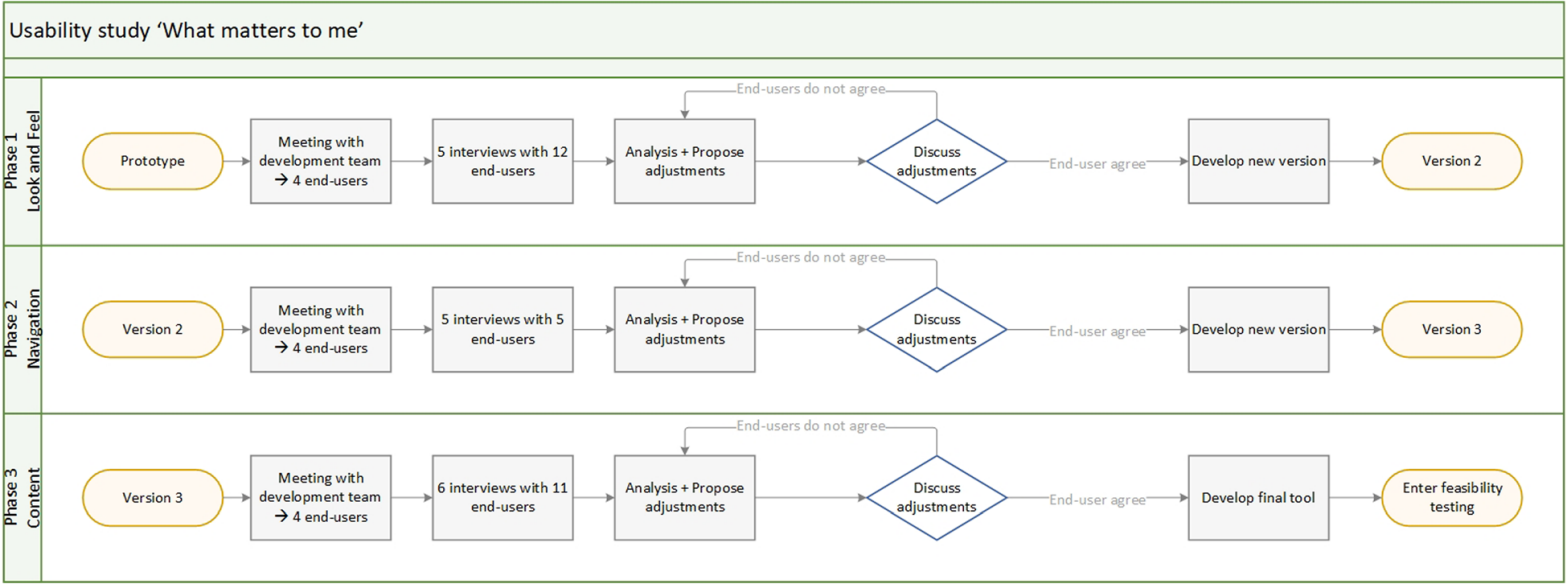
A flow diagram of the qualitative descriptive study ‘What matters to me’. The steps taken in the qualitative descriptive study during the three phases: Look and feel, Navigation, and Content. After each phase, a new version of ‘What matters to me’ was prepared based on the adjustments proposed by the end-users. The new version was used in the following phase of the study

This qualitative descriptive study applied a user-centred design allowing for an iterative process in which end-users influence how a tool takes shape [21]. The iterative process is a real-time evaluation of a tool, augmenting the competences of researchers with the knowledge of end-users [22]. The walkthrough methodology was used to test versions of the tool with a mutual shaping of social, cultural, and technical processes [23, 24]. A qualitative descriptive study was chosen to investigate the tool [25]. In line with studies of Savelberg et al. [26] and Garvelink et al. [27], the study consisted of three phases (Fig. 1):

- Look and feel: The look included the attractiveness and layout of the tool. The feel was the impression of a person on looking at and using the tool.
- Navigation: The navigation included the ease with which end-users went through the tool, and whether it was clear how to use the tool.
- Content: The content included the comprehensibility of all texts and propositions, the size of the tool, the relevance of included propositions, and possible missing propositions or information.

### Setting

This study considered clients in four long-term care sectors. Nine organizations were involved in three provinces of the Netherlands. These organizations provide care in settings including ambulatory, home care, and care in facilities such as nursing homes. Organizations included were one organization in the field of nursing and care for elderly, two in the care of people with disabilities, two in mental healthcare, one in social care, and three that provided services in all care sectors. The included professionals were independent care coordinators. They share knowledge about the route a client could follow during decision-making and the options a client might have, and they could support during consultations with care organizations.

### End-users

End-users were actually engaged in the decision-making process for long-term care or had recently chosen a care organization. The end-users were clients, relatives, and healthcare professionals. The inclusion criteria for users was the willingness to express an opinion about improvement of the tool and to share their experiences with long-term care. People without digital capabilities or understanding of the Dutch language were excluded [28], there were no exclusion criteria regarding demographics or cognitive abilities. Recruitment comprised two parts. First, four users were recruited to join a development team that was involved throughout the entire study. This team consisted of two clients and two professionals. Second, users were recruited at each phase for individual walkthrough interviews. Twelve users participated in five interviews in the Look and feel phase. Five users were consulted during five interviews in the Navigation phase, and the Content phase consisted of six interviews with eleven users. Different end-users were included in each phase.

Convenience sampling was applied to select the users from within the nine organizations included. The first four who responded to the recruitment letter formed the development team. If people were willing to participate, they received an information letter about the study and were telephoned by a researcher to explain about the project and verbal consent was given to set an appointment for the interview.

### Client participation

Client representatives, a development team, and individual users were involved in this study. The client representatives, from two independent care organizations in the Netherlands, were partners during the design phase to discuss all opportunities for client participation. Their role as partners also included writing information letters, recruiting users, reviewing topic lists, and being co-author. The members of the development team led the meetings and had the power of decision on the adjustments to the tool after each phase. The individual users were consulted during the interviews and informed about the findings and adjustments.

### Ethics

Ethical review and approval was obtained from the Medical Ethics Committee of Zuyderland Zuyd (17-N-79). The users gave written informed consent and were informed of their right to withdraw at any moment. Data were anonymized and data confidentiality was maintained.

### Data collection

Data were collected between November 2017 and March 2018 by means of meetings with the development team, walkthrough interviews with individual users, video-audio recordings, observations while users used the tool, and field notes. Every phase had a similar setup. First, a 90-min meeting was held with the development team, then individual walkthrough interviews were performed. These took the form of a step-by-step presentation of the tool in order to reveal experiences and thoughts of end-users. Interviews lasted between 30 and 60 min. One researcher arranged all the meetings with the development team and conducted the interviews. Meetings with the development team took place at a central location. A researcher visited the users who gave the interviews at home or at their care facility to ensure a trustworthy environment. Users with mental disabilities were accompanied by a caregiver. Video-audio recordings were made, and a researcher observed and made field notes.

A topic guide based on literature was developed for each phase and reviewed by client representatives [26, 27]. After each phase, a summarizing document containing all the feedback and comments of the users was compiled, including a proposal for adjustments to the tool. This document was mainly based on the field notes taken at the meetings with the development team and at the interviews. After agreement of all members of the development team, the adjustments were implemented, and a new version of the tool was created for the next phase.

#### Phase I – look and feel

The first version of the tool was a ten-page, picture-based tool to evaluate the look and feel. The content was determined by means of frameworks to assist preference elicitation, and a study into the decision-making process and preferences of clients in long-term care [14]. A literature overview of frameworks to elicit preferences in health and social care was performed. Five frameworks were combined and reflection meetings with four client representatives were held to obtain face validity. This resulted in five categories of preferences: family and friends, daily activities, living, finances, and health. This version contained the essential pages, including homepage, category page, proposition pages, pop-ups, and the overview (see Additional file 1). The pages were in a particular order, it was not possible to move back and forth.

All users involved in this phase were confronted with the tool for the first time during the interview, and were asked to click through the pages and talk about whatever came into their minds. Field notes were based on topics discussed during the meeting and interviews. The guide included topics to assess the feel, the look, and the general impression, To evaluate the feel, users were asked to express their impressions. To explore the look, users were asked to think aloud about the layout of the pages. The general impression was obtained by means of questions such as ‘What did you appreciate?’ and ‘What would you change to improve the tool?’ For continuing the second phase, adjustments were made to the first version based on the findings and approval of the end-users.

#### Phase II – navigation

The second version was an interactive web-based tool in which several elements were operational. It was possible to navigate back and forth through pages. Starting at the homepage, moving through the categories with the propositions, and continuing to the overview. The categories ‘family and friends’ and ‘finances’ were operational and could be filled-in on a 7-point Likert scale.

The members of the development team, who had already seen the first version, received a link to the tool and were asked to click through the category ‘family and friends’ before the meeting. The users who were interviewed individually did not receive the link beforehand to assess their first reaction. They were asked to click through some categories during the interview. A researcher took field notes, and observed the navigation. The topic guide of this phase focused on the ease with which users used the tool and their understanding of the path they should follow. The observations were used to gain further insight into the navigation. Following the interview, similar to the previous phase the general impression of the users was questioned. For continuation to the third phase, adjustments were made to the second version based on the findings and approval of the end-users included in the second phase.

#### Phase III – content

In the third version, all elements were operational and the users could test the content of the five categories. At the start of this phase, the development team received a link and were asked to click through all categories before the meeting. Other users were asked to click through and complete one or more categories during the walkthrough interview. Users were observed while they completed each category, and field notes were taken on their remarks about comprehensibility of texts and propositions The possible content of each category was discussed before answering the propositions. Users were motivated to comment on the relevance of the content and potentially missing propositions. Propositions that appeared irrelevant were discussed with other users during subsequent walkthrough interviews to investigate this further. Additional questions at the end of an interview included applicability to clients in long-term care, such as “How could this tool help people making decisions about long-term care?” and “How would you rate the tool?” After this phase, the adjustments were made to develop a final tool to be subsequently tested in a feasibility study.

Data saturation was determined per phase, according to the mathematical model that six users would uncover 80% of the usability problems [29]. In the first and second phases, saturation was reached after the meeting with the development team and five interviews, and in the third phase after the meeting with the development team and six interviews.

### Data analysis

All meetings and interviews were video-audio recorded and transcribed verbatim. Field notes and observations made during the interviews were combined with the transcripts. Content analysis was performed on all transcripts. During the preparation phase [30], both the deductive and inductive approaches were applied. The deductive approach was based on the main themes: Look and feel, Navigation, and Content. This was the start of the organising phase [30]. A matrix was developed comprising these three themes to analyse the data by content. The inductive approach was chosen to complement the organising phase with open coding in each theme and identification of categories.

Two researchers performed the deductive content analysis of three transcripts and compared the coding. There were no inconsistencies and one researcher continued the data gathering by content. Hereafter, the researchers performed the inductive content analysis of three transcripts and compared the open coding. The categories defined by the researchers were used to develop a coding sheet. One researcher continued the open coding using this sheet. The researchers discussed the coding and grouped all categories at the end of the organizing phase (see Additional file 2) [30]. Translation of the raw data took place in preparation of this study. NVivo11 software package was used for the data coding.

### Trustworthiness

Credibility was established by several procedures [31]. Method triangulation was done by multiple data collection methods, meetings with a development team, individual interviews, video-audio recording, field notes, and observations made during the use of the tool. Investigator triangulation was performed by a number of researchers who read, analysed, and compared findings. Peer debriefing took place at weekly meetings with the project team at which the scientific and organizational aspects were discussed. The summarizing document of each phase was part of the member check. At the end of the data collection, a member check was performed by means of an invitational conference for all users, organizations, and all those who took an interest. The findings were presented, and attendees tested and commented on the tool during this conference in order to validate it. A thick description was developed for the transferability [31], which included the sampling method, user selection, data collection, interviewing method, and analysis process. The user-centred design is a transferable method to be used in other web-based tool development contexts [31].

## Results

### End-users

Twenty-five users were included in this study, 52% male and 48% female, aged between 21 and 69 years. Sixty percent lived alone and the others lived with a partner or parents. Two users had never completed their education and ten had a low educational level. Twenty users were Dutch and five had other nationalities. Two users were from the nursing and care of elderly sector (two relatives), ten from the care of people with disabilities sector (one relative and nine clients), three from the mental healthcare sector (all clients), seven from the social care sector (all clients), and three from more than one sector (two healthcare professionals and one client).

The qualitative results are presented by the three phases consisting of an overview of the main findings in each category as defined during the analysis (Table 1). A description of the adjustments made after each phase is given after the findings.

**Table 1:** The categories defined in the three phases of the study, obtained from data analysis

Three phases	Categories of content analysis
Look and feel	Visualization
	First impression
	Layout
Navigation	Logic
	Accomplishing
Content	Comprehension
	Validity
	Practicality

### Phase I – look and feel

#### Visualization

Visualization consisted of colours, images/icons, and shapes. The colours were received very positively, but extra colours were requested, especially on the homepage.

*Client 5: “Looking at this colour here, the blue is very calming. You are not visually overwhelmed, which makes the page nice to look at and people will want to continue to read what it says.”*

The development team discussed the colours of the proposition pages in further detail. Every category of the tool was a different colour on the main page, but these colours changed on the proposition pages. The colour composition of each category had to become recognizable throughout the tool. Initially, the ‘finances’ category was green on the main page, but purple on the other pages.

**Fig. 2:**
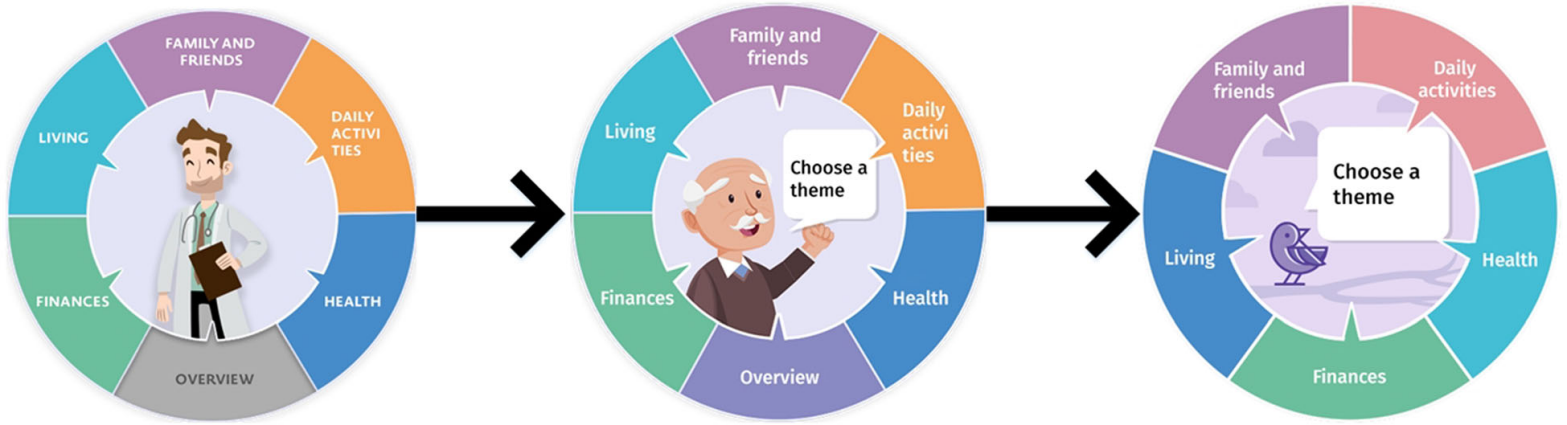
Development of the images used on the main page of the tool. On the left, the figure started as a person that looked like a doctor. The tool was not developed for doctors or for a hospital setting. Therefore, the suggestion was to change the image into a ‘normal’ person. The ‘older’ man in the middle figure was rejected because most clients could not identify themselves with this man. The last figure (on the right) was a bird. This is a more neutral image and was seen as a symbol of freedom

At the meeting with the development team, the images were considered to be too specific for the elderly. In the interviews all users made similar comments; they thought the images should be more neutral and applicable to all end-users. (see Fig. 2). The image of the doctor (on the left) was rejected as this tool was not for use in hospital settings. The tool was developed to support clients with their preferences construction in any care sector, therefore, a neutral persona was chosen. The man (image in the middle) was rejected because most clients could not identify with this man. Clients chose a bird (image on the right) as a neutral symbol, and some users compared this bird with freedom.

*Professional 1: “Considering the broad target audience, including older people, the elderly, children with a disability, anyone who can use a computer can use this. Then, the bird is a sympathetic symbol with which almost no-one will have negative associations. If you were to choose a person as a symbol, then it would always be the wrong person. A woman would be wrong because it was a woman, and a man would be wrong because it was a man. And if you were to give the woman or the man a colour, then this would be the wrong colour, especially nowadays.”*

*Client 2: “I associate a bird with freedom, the freedom to choose. I can identify with that thought.”*

*Professional 1: “Yes, neutral, freedom to choose, those feelings.”*

The users were motivated to discuss their perceived meaning of icons. Their thoughts were compared to the actual meaning of the icon. The icon for the category ‘activities’ was a calendar at first, but users associated this with time rather than activities, and some thought that it looked like an apartment building. In addition, the order of the icons appeared to be important, the first icon in the row belonged to the category ‘finances’, which was a euro sign, and gave one user the idea she had to pay to be able to use the tool.

*Client 4: “The icon with the euro sign gives me the impression I have to pay for this tool. I would change or replace this icon, because it is possible that I would not understand it and therefore not continue using this tool.”*

#### First impressions

The sub-theme first impressions involved the feeling and accessibility of the tool. The members of the development team felt comfortable, and agreed that the tool seemed accessible, friendly, and easy.

*Client 1: “I really like the lay-out of the website, its colours and the composition of the bird, it’s fantastic actually! It stimulates me continue to fill it in.”*

The feelings of other users were comparable. More negative feelings were expressed about the time indicator at the start of a category. This was intended to inform people about the potential time investment necessary to complete a category; the users agreed that it would induce time pressure instead of putting the user at ease.

#### Layout

The sub-theme layout comprised composition, font size, and form. The circular shape and the composition of the buttons to access different categories were considered pleasant and easy to use. However, the button to access the overview had to be separated from the circle because it was not a category where the users could answer propositions. The users considered the layout of the proposition pages well-ordered, and it was clear where to look.

*Client 8: “The page looks very well ordered. I love anything that is well organized. For me personally it is important that it is pleasant to work with and to look at. However, it is not just me. I think everyone would appreciate some organization in order not to become disoriented. We are disoriented enough to start with as we have already lost our homes, and if this tool was also chaotic we would probably say, ‘Sorry but we are not going to use this.’”*

They thought the understanding of the answer options for the 7-point Likert scale could be improved by numbering the answer options. They suggested the addition of the answer ‘not applicable’ would be beneficial and should be added to every proposition. The composition of the overview page caused difficulties in understanding where to focus due to the positioning of text blocks. Although the font size was not commented on by most users, some requested a button that could be used to increase the font size.

#### Adjustments to version I

The adjustments made to this version were based on the findings as presented above, and agreement of the development team. More colours were added to the homepage, and the colour composition of each category was consistently used throughout the tool. The images were changed to more neutral images (Fig. 2). The icons were changed in accordance with the suggestions of users to ensure comprehensibility and identification. Concerning, the positions of the icons, moving the euro symbol to the middle did not give the impression that the tool had to be paid for. The time indication at the start of a category was removed, the option ‘not applicable’ was added to every proposition, and the answer options were numbered. Although a button to increase font size was suggested, this was not included in the adjustments as this function is already available on digital devices. On the overview page, the configuration was changed to increase user-friendliness, and the button to access the overview became distinct from the circle (Table 2).

**Table 2:** Summary of adjustments

Phase	Adjustments
**Phase I (Look and Feel)**	- Change all pictures of the tool into more neutral pictures. - Colours: ○ More colour requested in the starting page (top picture). ○ The composition of colours more into one line over all pages of the tool. - Remove the time indication at the start of a category. - Add the option ‘not applicable’ to all propositions. - The button towards the overview needs to become more visible. - The configuration of the messages on the overview page need to change in order to make the messages more logical.
**Phase II (Navigation)**	- Visualization is necessary in order to scroll down on the homepage. - Make the accompanying texts shorter and more personal for the user. - The category money needs a revision: ○ What is the purpose of this category? More information is required. ○ New propositions such as “I want to know the costs for my care.” and “I need assistance with my administrative tasks.” - Clients ask for a read-aloud function. - The homepage should make clear how people who assist clients during the search should use the tool. - If someone finished one category, the explanation of the tool should change and include a suggestion to continue.
**Phase III (Content)**	- The language used should become as easy as possible. - Propositions: ○ More examples of answer are useful. ○ Similar propositions should be combined. ○ Ordering needs more logic. - Some aspects were missing: ○ Happiness and well-being “I need assistance to be happy.” ○ Children “When you have children: my children matter to me.” ○ Debts “Debts influence my life.” ○ Current living environment “I am happy at my own home.” - More information how to contact people who can assist with the search for long-term care (Independent Care Coordinators).

### Phase II – navigation

#### Logic

Logic covered ordering of pages, the clicks, and switching between pages. The development team and the other users thought that navigation was easy and the ordering of the pages was logical. When questioned, the users were positive about these aspects. Some navigational difficulties occurred, especially if users continued without reading a page or wanted to return to the homepage. The home button was a bird, similar to the bird that assists the users to answer propositions, but a house symbol as the home button was thought to be more familiar.

*Client 10: “It is very clear where to click, in this circle with the five categories. Also the bird, which appears and tells you how to continue. When you continue, this bar shows you the proposition you are at, and how many propositions will follow, and that is very convenient. As extra confirmation,, the category you are answering is written on top of the page. I thought this was very good.”*

Overall, users understood where to click, however, on the homepage it was not clearly visible that there was extra information about the tool below the start button. In addition, the information on the homepage was thought incomplete. Information about the use of the tool by informal caregivers was missing.

#### Accomplishing

This sub-theme included the accomplishment of tasks, comprehensibility of the navigational tools, whether users understood how to use the tool. The task was answering propositions from one or more categories.

*Client 12:* “*I think the propositions are very good - very easy -, I do not have to think hard about how to answer them. It is clear what I need to do and I can answer immediately.”*

The observations made clear that the users were able to complete the task without assistance. Navigation through the propositions and answering was easy. However, users found the assisting text blocks too long. The development team also considered these assisting text blocks as problematic for some clients, and a read-aloud function was recommended.

*Professional 2: “What strikes me is, I am stumbling over the word ‘articulate’. Taking the clients who will be using this tool into account, some words are too difficult. Will everyone understand what is meant? For example, this page ‘assists in articulating what matters most to you’. This sentence seems complicated and could also be shortened.”*

*Researcher: “Could you give us any tips?”*

*Professional 2: “Yes, I am on to it. I am writing some options and will come back to you in a moment.”*

*Client 1: “Maybe you could replace ‘articulating’ with ‘telling’? That would work better.”*

#### Adjustments to version II

**Fig. 3:**
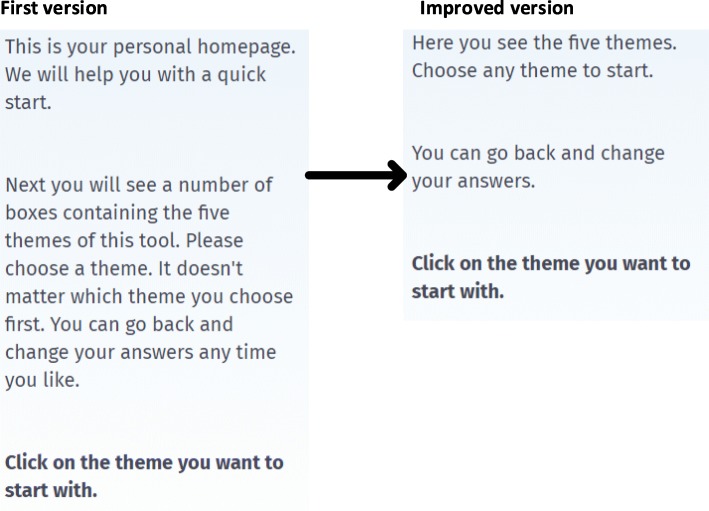
Development of the text block on the category page of the tool. Based on comments made by the end-users, the text on the right is shortened. Shorter sentences are made, each sentence starts on a new line, and more spaces are inserted

The text ‘return to home’ was added next to the bird to identify the home button, and on the homepage, a ‘more information’ button was added to show that further information was available. This section was updated with information for informal caregivers. Reviewing of the large text blocks was necessary, sentences were removed or shortened, and spaces were added in between lines (Fig. 3). In order to make texts accessible to users with lower reading capacities, a read-aloud function was included (Table 2).

### Phase III – content

#### Comprehension

The sub-themes of comprehension were language, texts, and category names. Different from the navigation phase, this sub-theme concerns the comprehension of all textual aspects, whether users understood the propositions and what their answers mean. Users found the meaning of the categories to be clear; the propositions were received well and regarded as being understandable. However, although understandable, the language used in the propositions was often difficult. It was suggested that combining similar propositions and adding example answers would improve the comprehensibility.

*Professional 2: “Considering the clients, we need to improve the content. For example, not only the language, but also order of the sentence. A sentence should be formulated as simply as possible, without sub clauses or auxiliary verbs, and everything aimed at the user. I think that is the way that most clients would understand.”*

#### Validity

Validity included missing or irrelevant propositions. Users were asked to formulate propositions they thought were missing, such as children, living with like-minded people, and well-being.

*Client 20: “A personal extra remark relating to the content and the propositions. What helps someone to become happy? There are no actual propositions on well-being. I do not know how to formulate this in a proposition but I’ll try, maybe something like ‘I want to do what makes me happy.’”*

When the users answered the propositions, they were asked which they considered irrelevant. The development team was critical of the category ‘finances’. They thought it too confrontational to ask whether someone could afford to spend more money on care. Propositions potentially missing from this category were also discussed. They suggested that the assistance clients need with finances and administration should be included. Other users had a different stance towards the category ‘finances’, they thought that the propositions were useful and suggested adding more propositions, such as assistance with debts.

#### Practicality

Practicality concerned the usefulness and possible use of the tool in practice. The members of the development team were enthusiastic when they saw that their suggestions had been applied in new versions. They agreed that the tool had improved a lot and that it could become a useful aid in conversations about long-term care. To increase the use of the tool in practice, information about people and organizations to contact for assistance with the search for long-term care would be beneficial.

#### Adjustments to version III

In improving, the focus was on easing the language and comprehensibility of propositions, by combining similar propositions and introducing the propositions in a logical order whereby similar topics follow each other. To ease reading, the questioning style of all propositions was unified, and to clarify the purpose of a proposition illustrative example answers were added under an information button. The category ‘finances’ was completely revised. Several proposition topics were added such as debts, children, and well-being. Extra information about organizations users could potentially contact for assistance in long-term care was added to the homepage. The last adjustments were implemented and the finalized tool was developed (Table 2).

## Discussion

The aim of this qualitative descriptive study was to understand the user requirements to develop a web-based preference elicitation tool for clients in need of long-term care. User requirements were elicited and inventoried in three phases. The feedback from the Look and feel phase was unequivocal, a pleasant layout and accessible tool. The most important adjustments were made to the images and the colour composition. Reducing the length of text blocks was the main adjustment that had to be made in order to improve navigation through the tool. During the Content phase, the language was discussed extensively and many suggestions were made. Another important adjustment was the addition of propositions considering well-being.

The user-centred design and the client participation applied in this study worked out as planned. All users were consulted during the data collection, most clients gave suggestions and advised the researchers how to improve the tool. The project team took this advice seriously. The comments provided by the end-users were directly translated into adjustments of the tool. The end-users were in the lead in deciding which adjustments were actually implemented in the tool, which placed the users a step higher on the participation ladder [32]. The plan was to involve all users in the analysis in a consultative manner. Just three interviewees made comments on the summaries of the findings and proposed adjustments, therefore, most users included in the individual interviews played a consultative role. The members of the development team followed the planned direction and took the final decision on approving suggested adjustments. During the conference, the users said they felt involved when making suggestions.

The tool was developed to answer the need for more assistance and understanding of preferences for long-term care. Clients and caregivers need more information and help to be able to increase the possibility of getting personal counselling [33]. In long-term care, clients are better supported by personal counselling than by a generic website only [34]. Personal counselling does not need to be a personal assistant, the help of a tool that is client-centred, supportive, and tailored to clients’ needs is also an option. McKenzie et al. [35] and Tinetti et al. [36] demonstrate that a tool to encourage person-centred planning could ensure that people have control over their lives in alignment with their priorities. There is a need for these tools, therefore, there are approaches to elicit preferences, but these have not been adopted in practice [2]. There are no supportive preference elicitation tools specifically for long-term care [37]. Tools that address a single disease show that people are better informed about options and what matters most, and they feel that they are participating in decision-making [9]. These single-disease oriented tools are unsuitable for helping clients to choose long-term care [9].

Preference construction and elicitation as part of collaborative deliberation is a communicative effort to understand priorities, preferences, and needs [5]. The communicative efforts could lead to create a plan consistent with someone’s preferences [38]. However, in order to talk about preferences, people need to be prepared [38]. Just asking seems too superficial, but little is known about how to help with these preparations. The tool designed in this study could increase this preparation. The goal of a preference elicitation tool is to provide the users with a limited set of propositions in order to explore their preferences in life and for care [39]. Therefore, this study aimed to include a minimal set of propositions. The Content phase showed that most propositions included in the tool were relevant to clients in need of long-term care. A greater emphasis on the propositions on assistance to increase well-being was requested. Although most suggestions concerned missing propositions, there were also irrelevant propositions, such as *‘If you were to draw a picture of something you really like, what would be in the picture?’* This proposition was considered strange or superfluous by more than half of the users.

The robust client participation during the development is a strength of this study. Including client participation in the study protocol appeared to be fruitful for the actual role of clients included in this study. Although strengths include the participation of a large number of people with a low educational level and the inclusion of clients from each sector, a weakness is the low participation rate of clients from the nursing and care of the elderly sector. Another strength of the study is the member checks, one after each phase and a fourth check at the end that took place at an invitational conference. The member checks were time consuming, but they ensured that the tool was actually adjusted in accordance with preferred user requirements. Although a strength is the data saturation, which was reached at each phase, data saturation of the entire study is debatable. It might have been beneficial to add a fourth phase to determine the stability of the coding sheet that was developed after the third phase, however, the development team approved all the findings and adjustments at the end of the third phase and during the invitational conference there were no new findings. The approaches described by Savelberg et al. [26] and Garvelink et al. [27] were combined and adapted to meet the aims of the web-based tool. Although this study was conducted in four long-term care sectors in the Netherlands, the study and the findings are generalizable to other settings. The need for more collaborative approaches, focus on client-centred care, and improving the client experiences are comparable [40–42]. Research into client-centred care is conducted in a large range of clinical contexts with a consensus that a broadly applicable model to assist with the communication would be feasible [40].

To investigate the use of this tool in practice, it will be necessary to perform feasibility tests in a follow-up study. Using the tool assists clients in eliciting their personal preferences by answering the propositions in preparation for a consultation with an independent care coordinator. The overview with answers of the propositions, presented on a printed version or an email to their independent care coordinator, can be used during these consultations to deliberate about the client’s personal preferences. In addition, family members can use this tool to support their loved ones or complete it on behalf of a very vulnerable loved one. An expectation of its use in practice is that independent care coordinators who assist during the decision-making process will explain about the use of the tool, motivate clients to use it, and to bring the resulting overview to a follow-up consultation. The independent care coordinator has access to the answers when provided (print-out or email) by a client. Currently, the tool is being implemented in long-term care organizations, but eventually this could be expanded to other areas, such as local authorities. Not every client will be in contact with an independent care coordinator, maybe they will only be in contact with their general practitioner, who could also motivate clients to use the tool. Before dissemination, exploration of its use in practice and feasibility is necessary. This tool may empower clients with the construction of preferences, but for most clients collaborative deliberation is also needed. The tool could assist deliberation and help healthcare professionals to find the best fitting care while taking their clients’ preferences into consideration.

## Conclusion

The user-centred design as presented in this study was fruitful for the understanding of user requirements as articulated by end-users. The intensive involvement of end-users resulted in improving each version of the tool. This study showed that the diverse populations of clients in long-term care were able to reach consensus about the five categories of preferences included in the tool. These categories made the tool human-centred instead of single-disease oriented. The expectation is that the tool ‘What matters to me’ will assist clients with preference elicitation, during consultations with professionals, and during their search for long-term care.

## Supplementary information

**Additional file 1: Figure S1.** Homepage of the tool used during the Look and feel phase of the usability study. **Figure S2.** Category-page of the tool used during the Look and feel phase of the usability study. **Figure S3.** Proposition-page of the tool used during the Look and feel phase of the usability study. **Figure S4.** Pop-up of the tool used during the Look and feel phase of the usability study. **Figure S5.** Overview of the tool used during the look and feel phase of the usability study.

**Additional file 2: Figure S6.** Deductive and inductive content analysis of the data obtained by the usability study. The analysis started with a deductive approach based on the three phases of the usability study. The categories within each phase were defined by an inductive approach.

## Data Availability

The dataset that support the findings and conclusion of this study are available from the corresponding author on reasonable request. The data are not publicly available due to privacy and/or ethical restrictions.
