# Supplementary material for: What matters to me – a web-based preference elicitation tool for clients in long-term care: a user-centred design
Source: BMC Med Inform Decis Mak. 2020 Mar 17;20:57. doi: 10.1186/s12911-020-1067-6 (PMC7077015; doi:10.1186/s12911-020-1067-6)
Supplement: Supplementary file 1 — Additional file 1: Figure S1. Homepage of the tool used during the Look and feel phase of the usability study. Figure S2. Category-page of the tool used during the Look and feel phase of the usability study. Figure S3. Proposition-page of the tool used during the Look and feel phase of the usability study. Figure S4. Pop-up of the tool used during the Look and feel phase of the usability study. Figure S5. Overview of the tool used during the look and feel phase of the usability study. [file 12911_2020_1067_MOESM1_ESM.docx]

**Additional file 1 – Characteristics of the tool**

This file shows the five essential pages of the tool, including the homepage, category page, proposition pages, pop-ups, and the overview. These essential pages were used during the Look and feel phase of this usability study. The homepage (Figure S1) contains several items informing the users about the purpose and use of the tool. The homepage also provides access to the preference elicitation part of the tool. The preference elicitation starts at the category page (Figure S2) where a user can choose a category to answer propositions. The propositions are all provided on separate pages (Figure S3). The users can click on the answers that fit to their opinion and continue with the following propositions. Sometimes pop-ups (Figure S4) are provided to give additional information, for example, when a user clicks further to see the overview but does not answer the propositions. The overview (Figure S5) is a list with all the answers the user has given to the propositions per category.


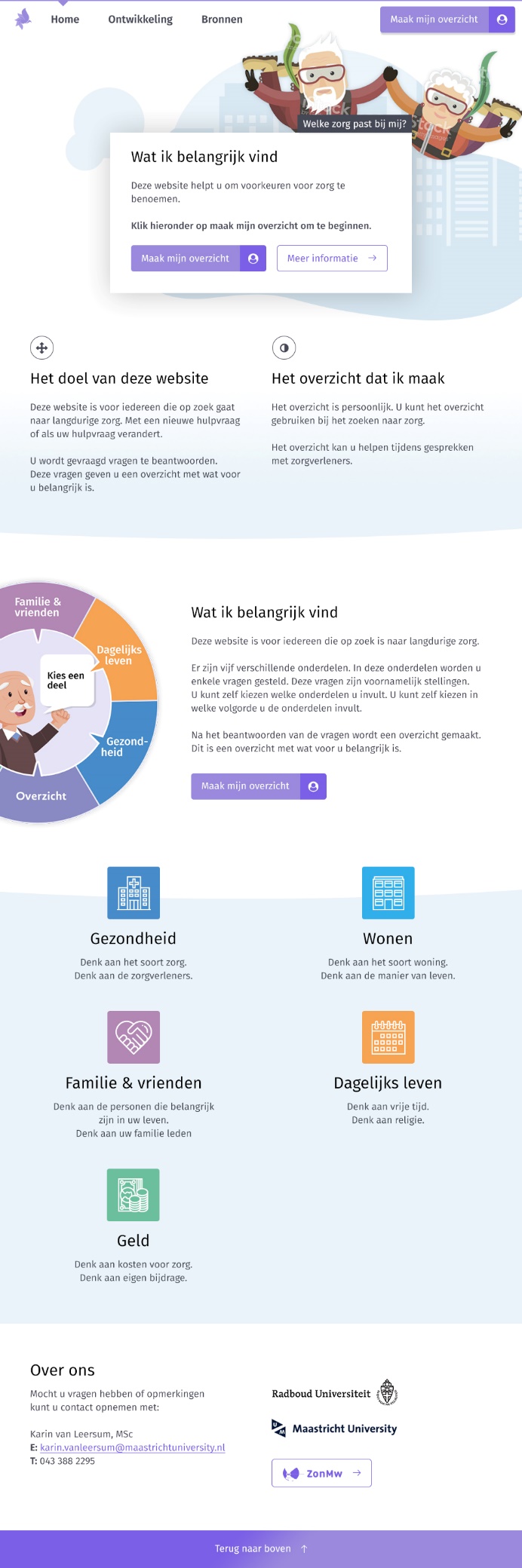


Figure S1. Homepage of the tool used during the Look and feel phase of the usability study.


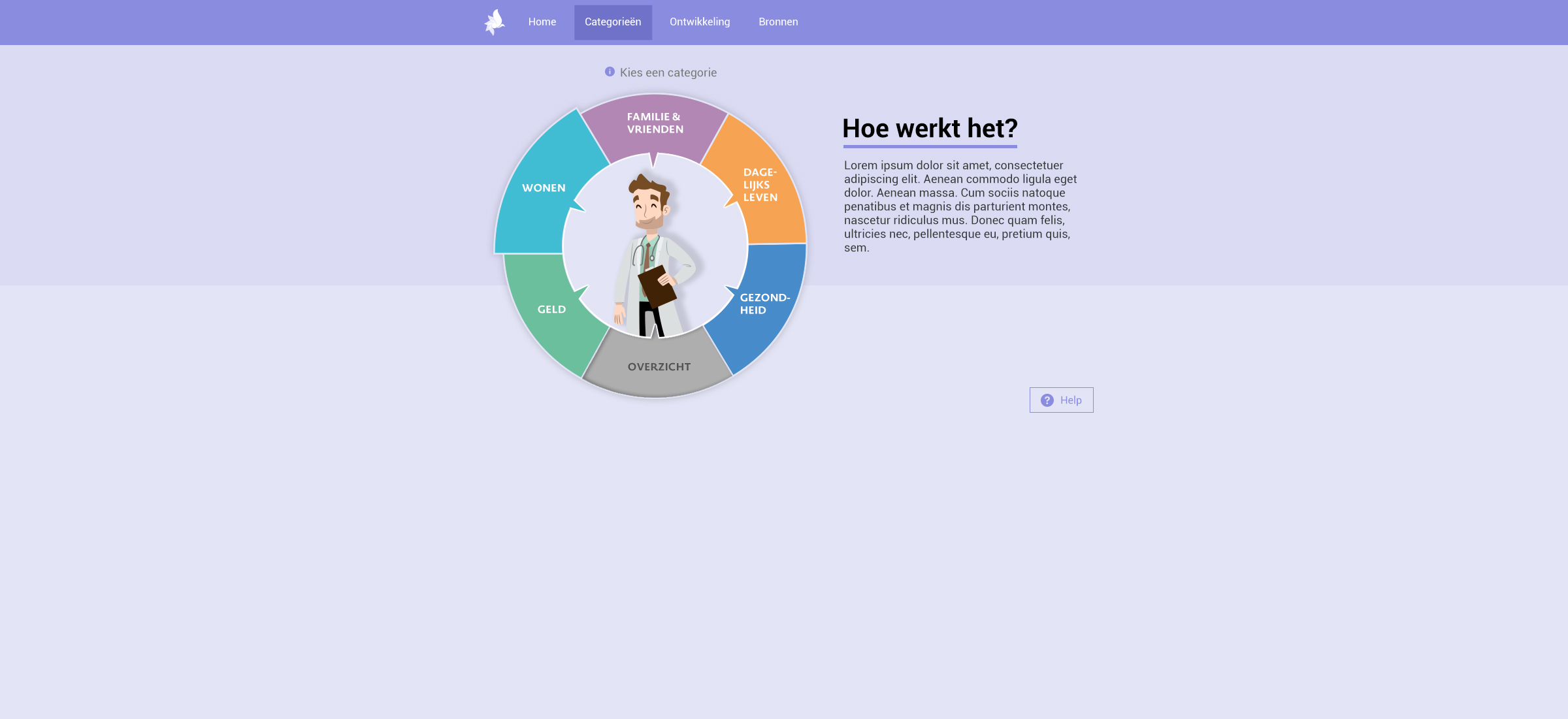


Figure S2. Category-page of the tool used during the Look and feel phase of the usability study.


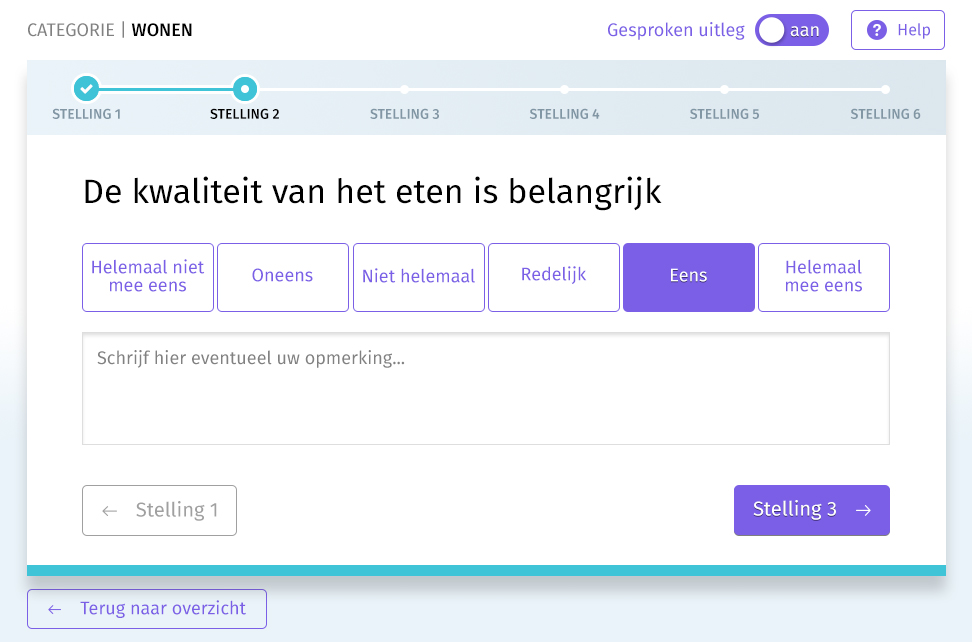


Figure S3. Proposition-page of the tool used during the Look and feel phase of the usability study.


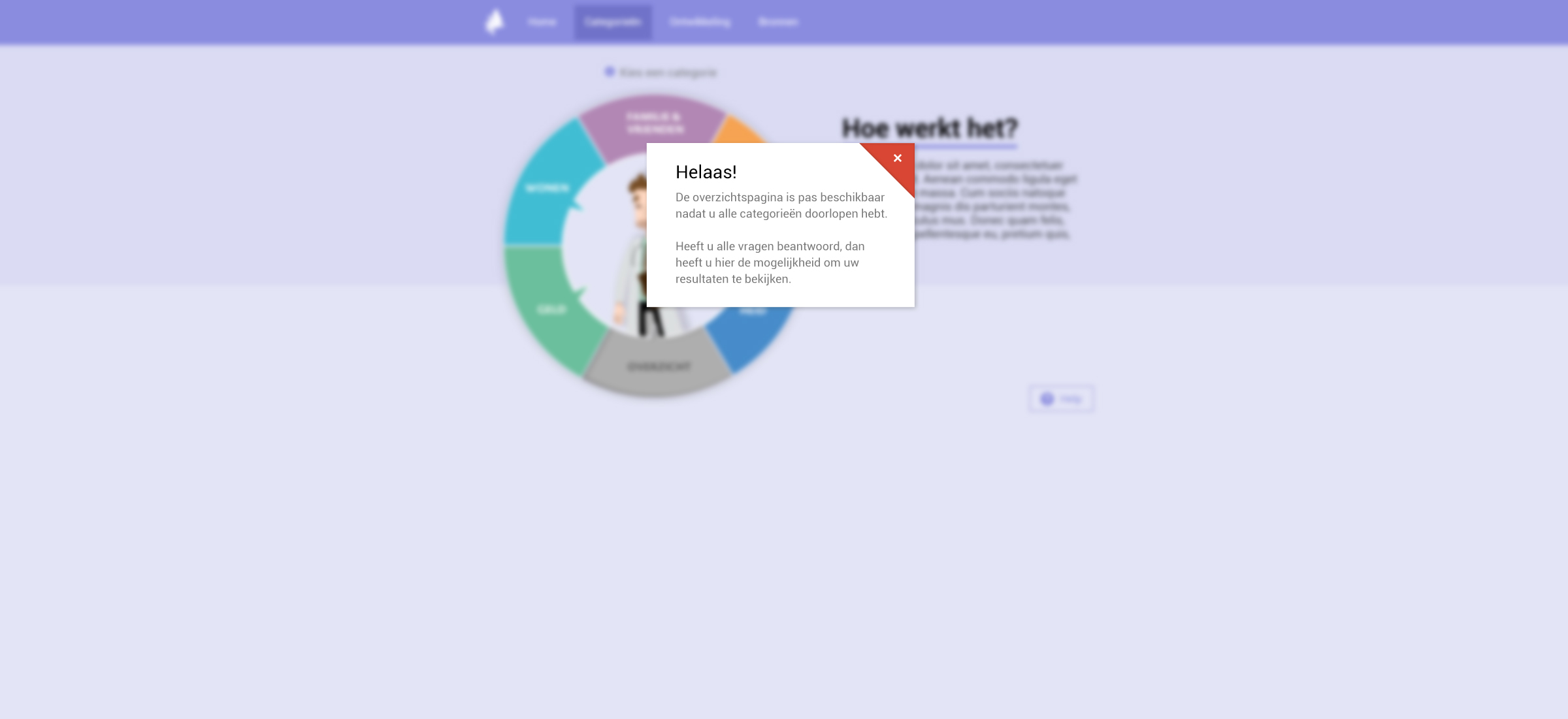


Figure S4. Pop-up of the tool used during the Look and feel phase of the usability study.


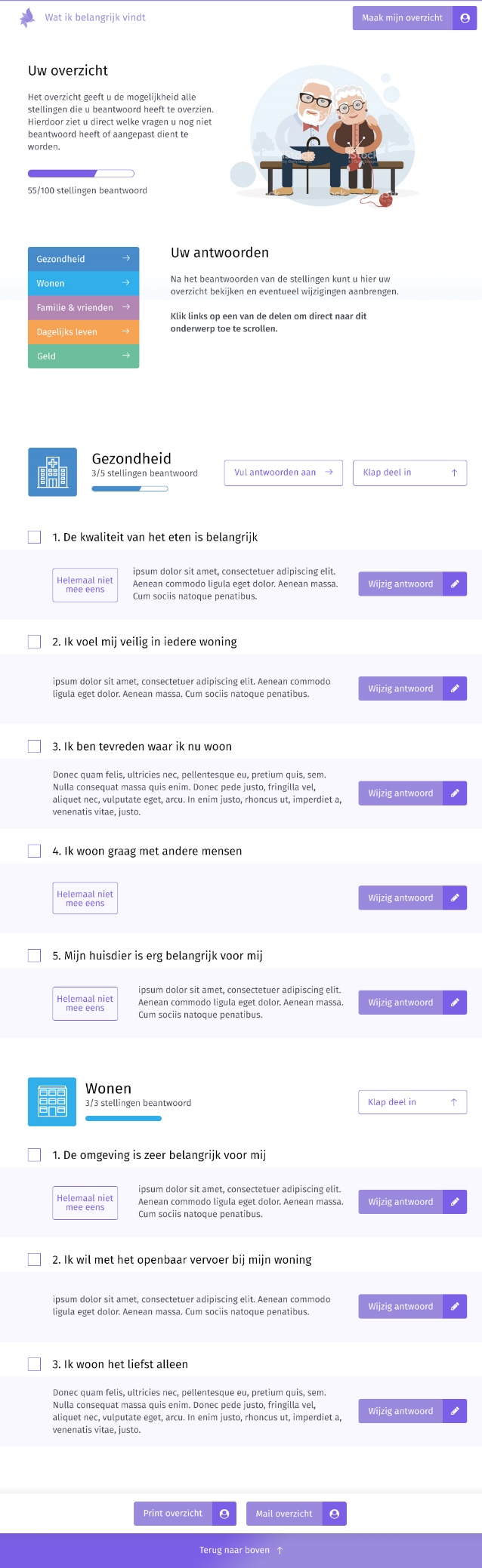


Figure S5. Overview of the tool used during the look and feel phase of the usability study.
