# Supplementary material for: What matters to me – a web-based preference elicitation tool for clients in long-term care: a user-centred design
Source: BMC Med Inform Decis Mak. 2020 Mar 17;20:57. doi: 10.1186/s12911-020-1067-6 (PMC7077015; doi:10.1186/s12911-020-1067-6)
Supplement: Supplementary file 2 — Additional file 2: Figure S6. Deductive and inductive content analysis of the data obtained by the usability study. The analysis started with a deductive approach based on the three phases of the usability study. The categories within each phase were defined by an inductive approach. [file 12911_2020_1067_MOESM2_ESM.docx]

**Additional file 2 – Categories of the content analysis**

The content analysis of this study consisted of a deductive and inductive approach. The deductive approach was based on the three phases of this usability study. After data gathering by content, the inductive approach was applied for the open coding and categorization. Figure s6 shows the categories used to report the analysing process and the results.

Figure S6. Deductive and inductive content analysis of the data obtained by the usability study. The analysis started with a deductive approach based on the three phases of the usability study. The categories within each phase were defined by an inductive approach.
